# *Toxoplasma gondii* Sporozoites Invade Host Cells Using Two Novel Paralogues of RON2 and AMA1

**DOI:** 10.1371/journal.pone.0070637

**Published:** 2013-08-05

**Authors:** Anna Poukchanski, Heather M. Fritz, Michelle L. Tonkin, Moritz Treeck, Martin J. Boulanger, John C. Boothroyd

**Affiliations:** 1 Department of Microbiology and Immunology, Stanford University School of Medicine, Stanford, California, United States of America; 2 Department of Pathology, Microbiology and Immunology, University of California Davis, School of Veterinary Medicine, Davis, California, United States of America; 3 Department of Biochemistry and Microbiology, University of Victoria, Victoria, British Columbia, Canada; Univ. Georgia, United States of America

## Abstract

*Toxoplasma gondii* is an obligate intracellular parasite of the phylum Apicomplexa. The interaction of two well-studied proteins, Apical Membrane Antigen 1 (AMA1) and Rhoptry Neck protein 2 (RON2), has been shown to be critical for invasion by the asexual tachyzoite stage. Recently, two paralogues of these proteins, dubbed sporoAMA1 and sporoRON2 (or RON2L2), respectively, have been identified but not further characterized in proteomic and transcriptomic analyses of *Toxoplasma* sporozoites. Here, we show that sporoAMA1 and sporoRON2 localize to the apical region of sporozoites and that, *in vitro*, they interact specifically and exclusively, with no detectable interaction of sporoAMA1 with generic RON2 or sporoRON2 with generic AMA1. Structural studies of the interacting domains of sporoRON2 and sporoAMA1 indicate a novel pairing that is similar in overall form but distinct in detail from the previously described interaction of the generic pairing. Most notably, binding of sporoRON2 domain 3 to domains I/II of sporoAMA1 results in major alterations in the latter protein at the site of binding and allosterically in the membrane-proximal domain III of sporoAMA1 suggesting a possible role in signaling. Lastly, pretreatment of sporozoites with domain 3 of sporoRON2 substantially impedes their invasion into host cells while having no effect on tachyzoites, and vice versa for domain 3 of generic RON2 (which inhibits tachyzoite but not sporozoite invasion). These data indicate that sporozoites and tachyzoites each use a distinct pair of paralogous AMA1 and RON2 proteins for invasion into host cells, possibly due to the very different environment in which they each must function.

## Introduction

Host cell invasion by Apicomplexan parasites, including *Toxoplasma gondii,* has generally focused on the asexual stages. It is a complex process, whereby the parasite invades the host cell in an active manner involving largely the use of parasite’s own machinery [1], [2] and a coordinated secretion of multiple proteins stored in at least two different organelles, the micronemes and the rhoptries [3], [4], [5], [6], [7], [8], [9]. Invasion begins with a tight attachment, reorientation (or high-affinity apical attachment) and the onset of gliding motility to help the parasite propel its way into the host cell. This latter step involves the formation of an intimate ring of attachment between the plasma membranes of the host cell and parasite [10], [11] that migrates down the length of the parasite as it invades. This transient structure is referred to as the moving junction (MJ; also sometimes referred to as the tight junction) and has multiple roles, including generating the parasitophorous vacuole (PV) [12] as the parasite pushes into the host cell.

In *Toxoplasma*, the MJ has been characterized extensively for tachyzoites, the rapidly-dividing, asexual form. The tachyzoite MJ is a multimeric protein complex known to be composed of Rhoptry Neck proteins (RON) 2, 4, 5, and 8 [3], [4] and Apical Membrane Antigen 1 (AMA1). RON2 has been predicted to span the host plasma membrane as it interacts with RONs 4, 5, and 8 on the host cytosolic side, and AMA1 on the parasite surface [13], [14]. AMA1, a type I transmembrane protein, is conserved across all Apicomplexans [15] and its knockdown has been shown to markedly reduce invasion [16]. Furthermore, blocking the AMA1 ectodomain either by antibodies or small peptides can inhibit invasion of host cells by both *Toxoplasma* and *Plasmodium* asexual stages [7], [17], [18], [19]. The intimate, high-affinity interaction of domain 3 of RON2 and the ectodomain of AMA1 is crucial for efficient invasion [13], [18] and structural analyses of the association for both *Toxoplasma* and *Plasmodium* asexual stages has shown an extensive, buried region of interaction between the two proteins [13], [20].

Contrary to the well-characterized tachyzoite invasion, very little is known about the mechanism of how *Toxoplasma* sporozoites invade. Sporozoites develop over the course of several days inside the oocysts that are shed by felids into the external environment. Upon ingestion by an intermediate host, sporozoites excyst and invade the host’s distal small intestine. At some point soon after the initial invasion, sporozoites convert into tachyzoites, which then disseminate throughout the host [21]. The exact mechanism of host cell invasion by sporozoites has not been studied but they have been reported to use a two-step process whereby invasion first produces a distended, primary vacuole from which the parasite then proceeds to elaborate a tighter, secondary vacuole in which it then grows [22]. The machinery used in these various steps has not been identified or investigated.

Recently, *Toxoplasma* sporozoites were subjected to detailed transcriptomic and proteomic analyses [23], [24]. It was found that, in addition to the well-characterized “generic” AMA1 and RON2, sporozoites also express two paralogues dubbed sporoAMA1 and sporoRON2, respectively, that are not expressed at detectable levels in tachyzoites or bradyzoites [24]. The identification of these paralogues drove the question as to the precise interactions and roles played by the two sets of AMA1/RON2 paralogues during sporozoite invasion. Here, we show that the generic and sporozoite-specific paralogues interact in a mutually exclusive manner. We also perform invasion inhibition assays with sporozoites and show that the sporoAMA1-sporoRON2 complex formation is critical for sporozoite invasion of the host cell, while the “generic” AMA1/RON2 interaction is dispensable for invasion of this lifecycle stage. Structural studies reveal the molecular basis for these observations.

## Results

### SporoRON2 and SporoAMA1 are Distinct from their “Generic” Paralogues

**Figure 1:**
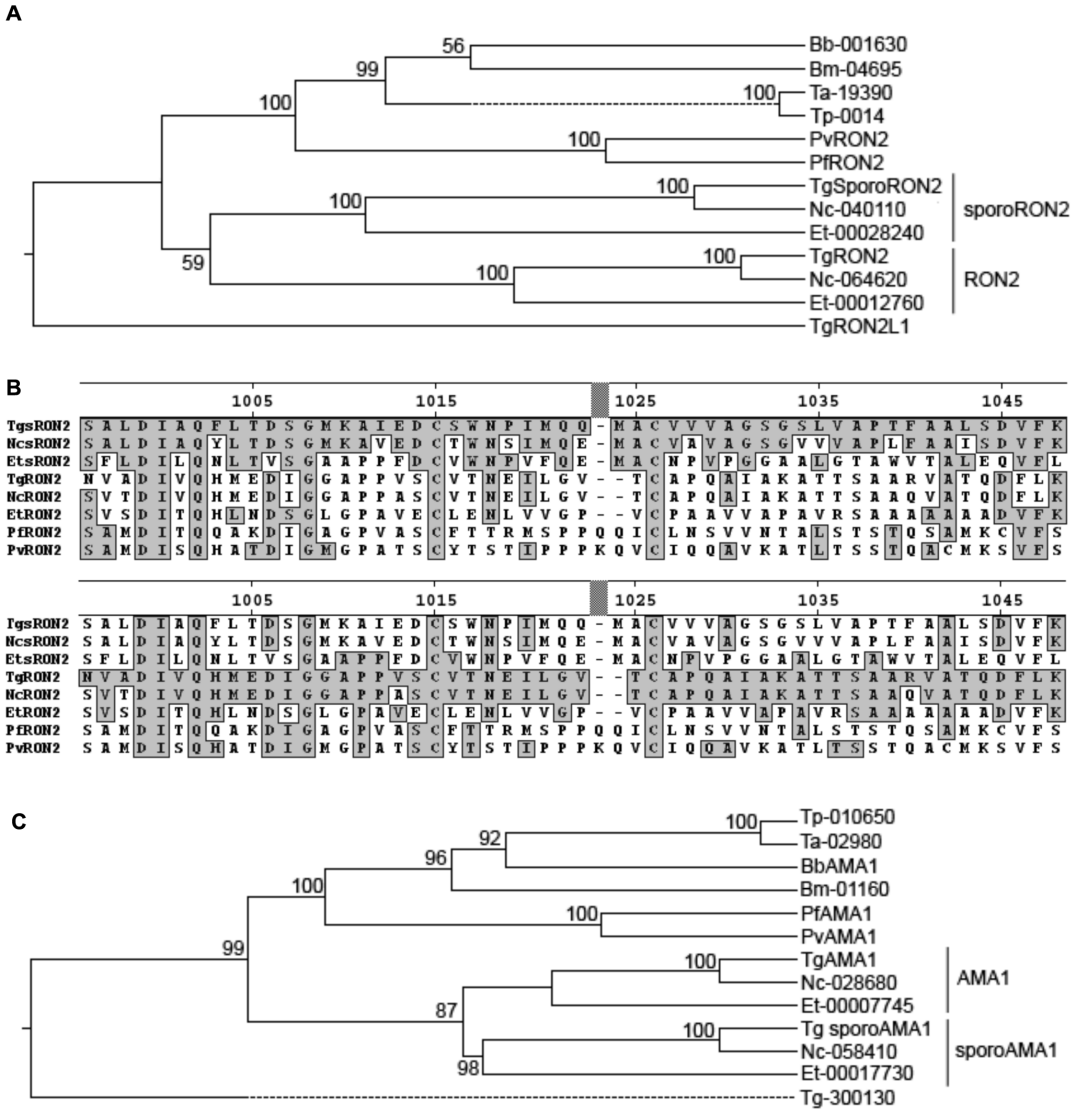
*T. gondii* sporoRON2 and sporoAMA1 are conserved in other *Apicomplexans* and are distinct from generic RON2 and generic AMA1. A. The *Toxoplasma* sporoRON2 polypeptide sequences were aligned with their respective homologues in *Eimeria tenella, Neospora caninum, Plasmodium* spp. (*P. falciparum* and *P. vivax*), *Babesia spp.* (*B.bovis* and *B.microti*), and *Theileria spp.* (*T. annulata* and *T. parva*) using ClustalW (as part of MegAlign software (Lasergene)) and an anchored phylogenetic tree was generated using the standard algorithm. The clusters including generic and sporozoite-specific versions of each protein are so-labeled. Bootstrapping analysis was performed to determine confidence intervals (1000 trials). B**.** Alignment of domain 3 (D3) of the indicated RON2 homologues was performed in ClustalW. Residues identical to that of *T. gondii* sporoRON2 are indicated with shading on the upper panel, while residues identical to those of *T. gondii* generic RON2 are boxed on the lower panel. Numbers indicate amino acid position of *T. gondii* sporoRON2 from the starting Methionine. C. As for (A) except using the predicted sporoAMA1 polypeptide sequences.

The existence of sporozoite-specific versions of “generic” RON2 and “generic” AMA1 in *Toxoplasma* sporozoites begs the question of their role in sporozoite invasion. To address this, we first asked how prevalent are these proteins in related parasites with similar life cycle stages. This was done by creating a rooted phylogenetic tree using ClustalW algorithms on the full amino acid sequences of the RON2 homologues present in these related species. As seen in Figure 1a, *Neospora* and *Eimeria* have orthologues of *Toxoplasma* sporoRON2 that segregate in a distinct and separate clade from the orthologues of the original *Toxoplasma* generic RON2 in these species. Only a single RON2 homologue is present in the representative *Plasmodium*, *Babesia* and *Theileria* species examined and the clade that includes these latter RON2 sequences is distinct from both the generic RON2 and sporoRON2 clades seen with the three *Eimeriorina* (*Toxoplasma*, *Eimeria* and *Neospora*). This suggests that the duplication that led to the two RON2 clades in the Eimeriorina occurred after its split from the Haemospororina (including *Plasmodium, Babesia and Theileria*). This is consistent with the fact that the two RON2 versions in the Eimeriorina appear equally closely related to the single RON2 in the Haemospororina. To further elucidate the differences between these proteins, we aligned the critical domain 3 of representative generic RON2 and sporoRON2 orthologues (Fig. 1b). This domain of generic RON2 has been previously shown to interact with generic AMA1 in a critical step for invasion [18]. As seen in Figure 1b, the clustering of the RON2-like sequences in the other species apparent at the whole protein level plays out similarly for the crucial domain 3. In fact, for species like *Toxoplasma gondii* that have generic and sporozoite-specific paralogues, there is very little conservation between the two at the individual amino acid level, with a few notable exceptions including a pair of cysteines that are known for generic RON2 to form an intramolecular disulfide bond [13].

Similar analyses were performed for the AMA1 homologues in these species. Construction of an anchored tree revealed a similar distribution of clades, with one set of AMA1 homologues in *Toxoplasma*, *Eimeria* and *Neospora* forming a distinct clade that includes sporoAMA1 and another that includes the generic AMA1 of *Toxoplasma* (Fig. 1c). The *Plasmodium*, *Babesia* and *Theileria* species analyzed have only a single AMA1 homologue each and these form a separate clade that is equally closely related to the generic and sporoAMA1 clades of the Eimeriorina. The existence, however, of an orthologue of sporoAMA1 in the genera having orthologues of sporoRON2 supported the hypothesis that these sporozoite-specific versions of a well-described generic pairing might themselves be specifically interacting.

### SporoRON2-D3 Forms a Specific Interaction with sporoAMA1

**Figure 2:**
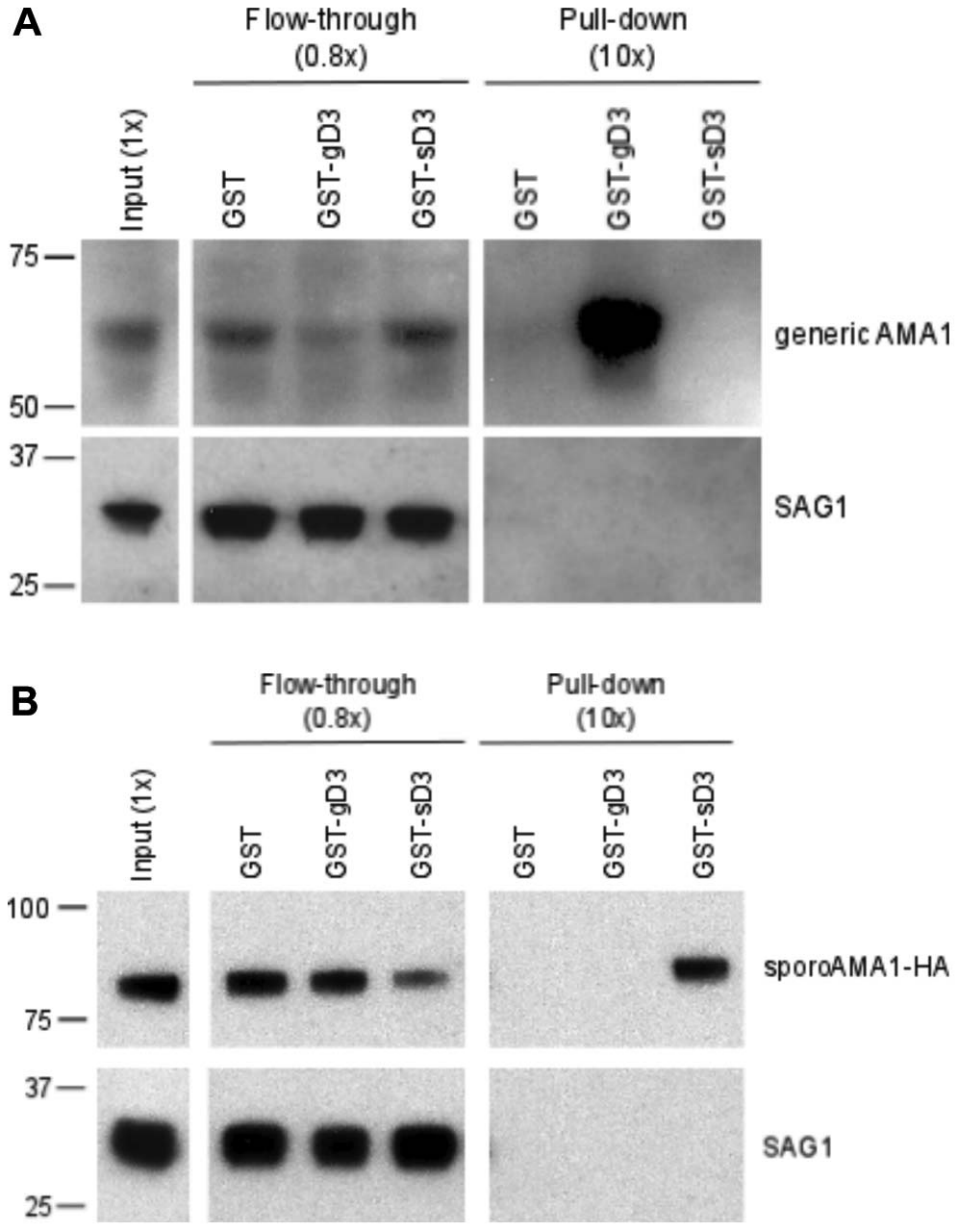
The sporo- and generic versions of RON2-domain 3 interact only with their respective sporo- and generic AMA1 partners. A. Molar equivalents of GST, GST-gD3, or GST-sD3 were added to lysates of RHΔ*hxgprt* and after NP-40 solubilization the material that did not bind to the GST fusions (“flow-through”) or that was pelleted with the fusions (“pull-down”) was resolved by polyacrylamide gel electrophoresis and analyzed by immunoblotting with antibodies to generic AMA1 or SAG1 as a control for loading and nonspecific pelleting. Parentheses indicate parasite equivalents of the different fractions relative to input (1×). Size markers indicated in kDa. B. GST-pull-down experiments were performed as described in (A) except using RHΔ*hxgprt* that were transiently expressing sporoAMA1-HA and the sporoAMA1 was detected using antibodies to the HA-epitope tag.

It has previously been shown that there is an extensive and tight interaction between generic AMA1 and domain 3 of generic RON2 [9], [13], [18]. As the sequences of domain 3 of generic RON2 (gD3) and sporoRON2 (sD3) differ substantially (Fig. 1b), we predicted that sD3 would show little if any ability to interact with generic AMA1. To test this prediction, we generated a fusion of sD3 (amino acids 995 to 1048) with Glutathione-S-transferase (GST). In parallel, we prepared a GST fusion of gD3 (amino acids 1293–1345), as previously described [18]. Tachyzoite lysates were mixed with these and glutathione-coupled beads were used to capture parasite proteins that bound to each. The results showed that, as previously reported, generic AMA1 was specifically enriched in the eluate fraction incubated with GST-gD3 but no such binding was observed with GST-sD3 or GST (Fig. 2a). These data indicate that, as predicted from its sequence divergence, GST-sD3 has little if any ability to specifically interact with generic AMA1.

The existence of a sporozoite-specific version of AMA1 suggested this might be the binding partner of sporoRON2. To test this, we repeated our GST pull-down experiment. This time, however, tachyzoites were transiently transfected with a plasmid containing full-length sporoAMA1 with a C-terminal HA tag. After allowing the transfectants to grow for 48 hours, the parasites were lysed and GST, GST-sD3 and GST-gD3 used for affinity purification. Input, the flow-through and the eluate were analyzed by immunoblotting for the presence of the HA-tagged sporoAMA1 (Fig. 2b). The results show sporoAMA1 was specifically enriched in the eluate fraction of GST-sD3, and not in the eluates of GST or GST-gD3 indicating that sporoRON2 and sporoAMA1 do indeed form a specific interaction, independent of their generic paralogues.

### SporoAMA1 Presents an Extensively Guarded Apical Surface

**Figure 3:**
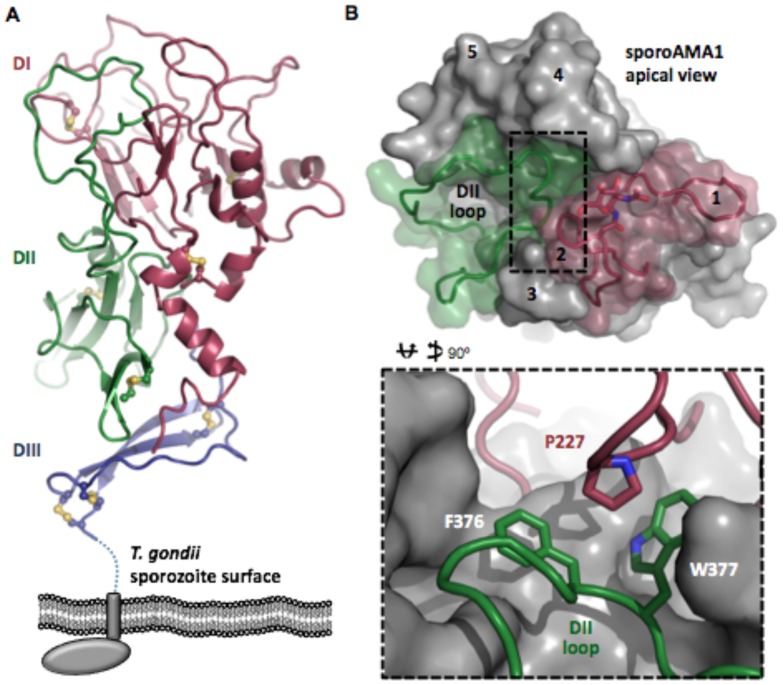
SporoAMA1 presents a highly guarded apical groove. A. Stacked three domain architecture of sporoAMA1 shown in the predicted organization to the *T. gondii* sporozoite plasma membrane with the three ectodomains indicated as DI in burgundy, DII in green and DIII in blue. Disulfides are shown as yellow sticks. Dotted line indicates extended Pro/Glu rich region between the conserved portion of DIII and the transmembrane domain (grey rectangle) that leads through to the C-terminal domain (grey oval). B. Apical view of apo sporoAMA1 structure, with core structure shown as grey surface and DI surface loops that guard the apical groove shown as burgundy cartoon and semi-transparent surfaces, and the DII loop as a green cartoon and semi-transparent surface. N-linked glycosylation on Asn230 shown as sticks. Numbers indicate surface loops that frame the apical groove. Inset: sporoAMA1 DII loop residues Phe376 and Trp377 (green) are pinned into the apical groove by Pro227 at the tip of loop 2 (burgundy).

The structure of generic AMA1, co-crystallized with a synthetic peptide representing generic RON2 domain 3 (TgRON2 synthetic peptide (sp)) has previously been reported [13]. To better understand the mutually exclusive nature of the interaction between the sporozoite and generic AMA1/RON2 pairings, we set out to solve the crystal structure of apo sporoAMA1 and of sporoAMA1 bound to a portion of sporoRON2-D3 (sD3). Overall, the structure of apo sporoAMA1 conforms to the expected three-domain architecture observed for other AMA1 structures (Fig. 3A) [20], [25], [26], [27], but reveals several divergent substructures including a network of apical surface loops that provide an unexpected degree of integration with the apical groove (Fig. 3B). In particular, apical loops 1 and 2 of domain I and the extended loop of domain II (DII loop) cover the region occupied by RON2 in previously determined AMA1/RON2 co-structures from both *T. gondii* and *P. falciparum* (Fig. 3B) [13], [28]. In addition, sporoAMA1 loop 2 is slightly longer than the analogous loop in generic AMA1 and has an N-linked glycosylation at Asn230 that is positioned atop the groove (Fig. 3B; note that while generic AMA1 has been reported to be naturally N-glycosylated in tachyzoites [29], no information exists on sporoAMA1 in this regard but it does have a consensus N-linked site which is efficiently N-glycosylated in the insect cells used to generate the recombinant sporoAMA1 studied here). Loop 2 folds in over the top of the groove, and positions Pro227 directly over the tip of the DII loop, which appears to compensate for the lack of the central anchoring tyrosine observed in generic AMA1 (sporoAMA1 Ser252; generic AMA1 Tyr230) [25], with Pro227 effectively pinning DII loop Phe376 and Trp377 into the base of the apical groove (Fig. 3B inset). Based on the degree to which the apical groove of sporoAMA1 is guarded, numerous specific interactions between sporoAMA1 and sporoRON2, particularly in the predicted initial binding site at the cysteine loop, are likely to be required to overcome the energy barriers of DI apical loop rearrangement and displacement of the DII loop.

### The Apical Groove of sporoAMA1 is Specific for sporoRON2-D3

**Figure 4:**
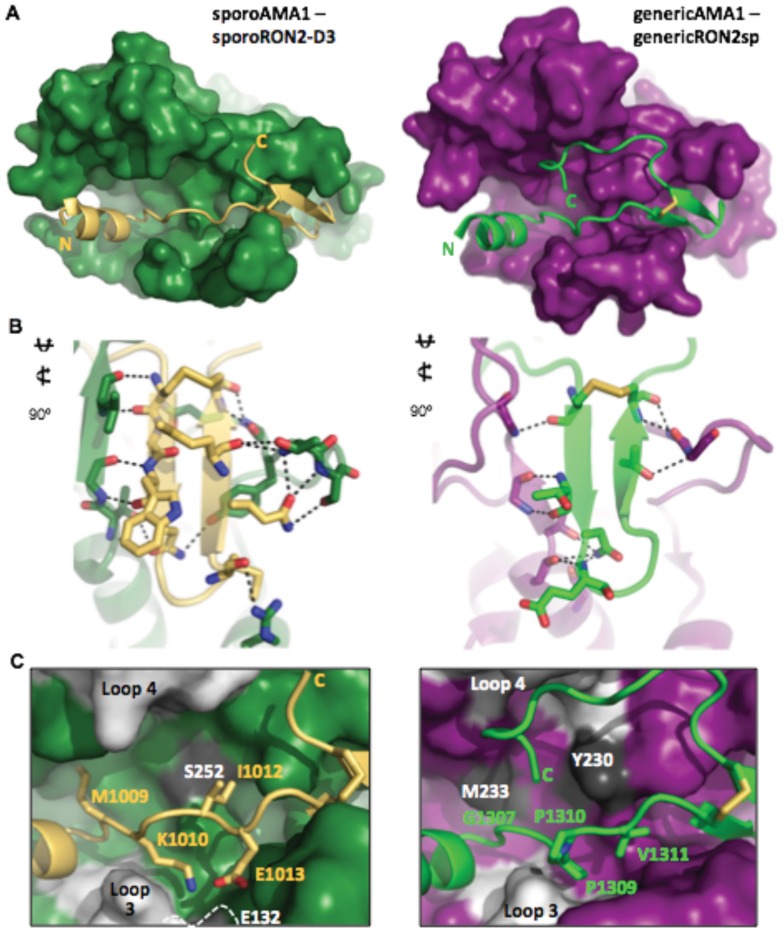
Specificity between SporoAMA1 and SporoRON2-D3 is achieved through interactions within both the cysteine loop and the connecting coil. A**.** Apical view of sporoAMA1 (green surface) bound to sporoRON2-D3 (gold cartoon) (left), showing conservation of the overall AMA1/RON2 binding paradigm with generic AMA1 (purple surface) – generic RON2 synthetic peptide (sp; green cartoon) (right; PDB 2Y8T). Note that the extreme C-terminal portion of the sporoRON2 peptide is disordered as a result of its relatively “early” exit from the stabilizing environment of the hydrophobic groove and therefore is not resolved in this structure. B. Cysteine loop interactions clearly differ between sporoAMA1-sporoRON2-D3 (left) and generic AMA1-generic RON2sp (right). Hydrogen bonds shown as dotted black lines. Colored as in (A). C. Additional specificity is gained through interactions with sporoAMA1 (green surface) or generic AMA1 (purple surface) and the RON2 coil that connects the N-term helix to the cysteine loop. Central groove residue (sporoAMA1 Ser252 or generic AMA1 Tyr230) and generic AMA1 groove residue Met233 colored dark grey. Beta-hairpin loop 3 and variable loop 4 colored light grey. Side chains of sporoRON2-D3 (gold cartoon) and generic RON2sp (green cartoon) involved in specificity shown as sticks.

The co-structure of sporoAMA1 with sporoRON2-D3 reveals significant complementarity, with a buried surface area of 3274 Å^2^, 23 intermolecular hydrogen bonds (Table 1), and a complexation significance score of 1.00 indicating physiological relevance [30]. SporoRON2-D3 is integrated into the apical groove of sporoAMA1 through an N-terminal α-helix seated in the area exposed by displacement of the domain II loop and connecting coil ordered through the center of the groove to the disulfide-bound beta hairpin loop at the opposite end of the apical surface (Fig. 4A left). This organization mimics the overall binding paradigm previously observed for generic AMA1/RON2 complexes from *T. gondii* tachyzoites (Fig. 4A right) and *P. falciparum* [13], [28].

**Table 1:** Hydrogen bond interactions observed in the sporoAMA1-sporoRON2-D3 (reported here) and generic AMA1-RON2sp (PDB 2Y8T; chains A and B, respectively) co-structures, aligned based on RON2 sequences with similar interactions bolded and the cysteine loop region residues are identified by italicized text.

sporoRON2-D3	sporoAMA1	Distance (Å)	genRON2sp	genAMA1	Distance (Å)
			Glu1303 [Oε1]	Gln361 [Nε2]	2.55
			Glu1303 [Oε2]	Arg111 [N]	3.46
**Asp1006 [O]**	**Arg135 [NH1]**	**3.54**	**Asp1304 [O]**	**Arg111 [NH1]**	**2.98**
Asp1006 [Oδ1]	Arg135 [NH1]	3.86	Asp1304 [Oδ1]	Gln361 [Nε2]	2.75
Asp1006 [Oδ2]	Ser383 [N]	3.78			
**Gly1008 [O]**	**Ala255[N]**	**3.51**	**Gly1306 [O]**	**Met233 [N]**	**3.65**
			Val1311 [N]	Tyr230 [OH]	3.54
Glu1013 [O]	Gln183 [Nε2]	3.04	Val1311 [O]	Tyr230 [OH]	2.56
Asp1014 [O]	Gln183 [Nε2]	3.23			
*Cys1015 [N]*	*Val225 [O]*	*3.35*			
***Cys1015 [O]***	***Val225 [N]***	***3.06***	***Cys1313 [O]***	***Met204 [N]***	***3.06***
*Ser1016 [N]*	*Gln183 [Oε1]*	*3.44*			
*Ser1016 [Oγ]*	*Gln183 [Oε1]*	*2.32*			
***Trp1017 [N]***	***Ile223 [O]***	***2.75***	***Thr1315 [N]***	***Val202 [O]***	***2.90***
***Trp1017 [O]***	***Ile223 [N]***	***2.96***	***Thr1315 [O]***	***Val202 [N]***	***2.85***
*Asn1018 [Nδ2]*	*Tyr185 [OH]*	*3.46*	*Asn1316 [Nδ2]*	*Phe197 [O]*	*3.47*
*Asn1018 [Oδ1]*	*Thr222 [N]*	*3.52*	*Asn1316 [Nδ2]*	*Lys200 [O]*	*3.70*
*Asn1018 [Oδ1]*	*Thr222 [Oγ1]*	*3.59*	*Asn1316 [Nδ2]*	*Thr201 [Oγ1]*	*2.76*
			*Glu1317 [N]*	*Lys200 [O]*	*2.99*
*Met1021 [O]*	*Arg202 [NH2]*	*3.47*			
*Gln1023 [Nε2]*	*Ser187 [Oγ]*	*3.69*			
*Gln1023 [Oε1]*	*Thr186 [N]*	*3.49*			
*Gln1023 [Oε1]*	*Ser187 [N]*	*3.08*			
*Met1024 [O]*	*Thr186 [N]*	*3.28*			
*Met1024 [O]*	*Thr186 [Oγ1]*	*3.65*			
			*Thr1322 [Oγ1]*	*Thr165 [N]*	*3.59*
***Cys1026 [N]***	***Val184 [O]***	***2.69***	***Cys1323 [N]***	***Val164 [O]***	***2.92***
***Cys1026 [O]***	***Val184 [N]***	***2.82***	***Cys1323 [O]***	***Val164 [N]***	***2.94***
			Gln1326 [Nε2]	Thr144 [Oγ1]	3.69
			Ala1327 [O]	Glu145 [N]	2.87
			Ala1329 [N]	Pro143 [O]	2.97
			Lys1330 [N]	Glu145 [Oε1]	3.16
			Ala1331 [O]	Trp253 [Nε1]	3.35
			Thr1333 [O]	Tyr230 [OH]	2.90
			Thr1333 [Oγ1]	Tyr230 [OH]	3.70

Despite the similar overall binding paradigm, a detailed analysis of the interactions between each paralogue pair revealed the basis for the biochemically observed specificity. While cross-genera specificity of the AMA1/RON2 interaction has been mainly attributed to interactions within the cysteine loop [13], [28], between the *T. gondii* paralogue pairs there appear to be major contributions to specificity from both the cysteine loop and the coil connecting the N-terminal helix to the cysteine loop. Nearly three quarters of the hydrogen bonds formed between sporoAMA1 and sporoRON2-D3 are found within the cysteine loop region, and only the strictly backbone interactions are observed at the generic AMA1-generic RON2sp interface, which contains approximately half the number of hydrogen bonds observed for generic AMA1 and the generic RON2 cysteine loop (Table 1; Fig. 4B). Moving outward from the cysteine loop region, the coil connecting the N-terminal helix to the cysteine loop has three notable shifts in the intermolecular interface between the paralogue pairings (Fig. 4C). Firstly, sporoRON2 Met1009 buries into a deep cleft in the side of sporoAMA1, while the corresponding residue in generic RON2 is Gly1307 and there is no cleft in this region of generic AMA1 due to the presence of Met233 (Fig. 4C). Secondly, beta-hairpin loop 3 is four residues shorter in sporoAMA1 than generic AMA1, which together with the introduction of a glutamic acid residue in this loop (sporoAMA1 Glu132), presents an enlarged pocket with sufficient shape and charge complementarity to accommodate an extended lysine residue of sporoRON2 (sporoRON2 Lys1010) (Fig. 4C left). In contrast, other AMA1/RON2 co-structures present a shorter AMA1 pocket accommodating a compact RON2 proline (generic *Tg*RON2 Pro1309; *Pf*RON2 Pro2033) (Fig. 4C right). Finally, the flattened surface caused by the presence of groove-central Ser252 in sporoAMA1 readily accommodates a bowed out conformation of sporoRON2-D3 consisting of Ile1012 and Glu1013, whereas the corresponding central residue in generic AMA1, Tyr230, limits the accessible area in this region and can only accommodate a valine (Val1311) flipped down towards the base of the groove and held in position by Pro1310 (Fig. 4C). All three of these interactions clearly show receptor-ligand co-evolution that provides specificity for the generic and sporozoite-specific AMA1/RON2 pairs of *T. gondii*.

### The C-terminal Region of SporoRON2-D3 is not Required for Coordination

While some differences between the paralogue pairings contribute to specificity, others are likely the result of capturing different biologically relevant conformations. The C-terminus of sporoRON2-D3, for example, does not extend the length of the groove as observed in the generic AMA1-RON2sp co-structure, but rather exits the groove shortly after the disulfide of the beta hairpin loop. This conformation of sporoRON2-D3 likely results from sporoAMA1 loop 4 being four residues shorter than the corresponding helical backstop of generic AMA1, which results in an approximately 5Å encroachment of the loop towards the center of the groove (Fig. 4C). SporoAMA1 loop 4 shows clear mobility in the crystal structure, and combined with the flexibility inherent to the C-terminal sequence of sporoRON2-D3 (CVVVAGSGS) suggests that two conformations of the complex likely exist in solution: first, AMA1 loop 4 is directed towards the groove center and the RON2-D3 C-terminus exits the groove, and second with AMA1 loop 4 displaced from the groove center and the RON2-D3 C-terminal sequence threading back through the groove, as seen for generic RON2sp. This rationale is supported by a comparison with the two previously determined structures of *Pf*AMA1 in complex with *Pf*RON2 peptides of different lengths; in the *Pf*AMA1-*Pf*RON2sp1 co-structure, *Pf*AMA1 loop 4 is displaced from the groove and the *Pf*RON2sp1 C-terminal region threads back through the groove similar to generic RON2sp, while in the *Pf*AMA1-*Pf*RON2sp2 co-structure, *Pf*AMA1 loop 4 is shifted about 4 Å towards the groove center and the two post-cysteine loop residues modeled appear to exit the groove in the same fashion as sporoRON2-D3 [28]. In addition, the way that sporoRON2-D3 forms contacts on both edges of the groove (Met1009 and Lys1010; Fig. 4C) may explain why it does not require coordination of the post-cysteine loop region for binding, and provides an interesting comparison with the recently determined *Pf*AMA1-R1 structure, where trans-groove contacts participate in coordination of the linear R1 peptide [28].

### Localization of sporoAMA1 and sporoRON2 in Sporozoites

**Figure 5:**
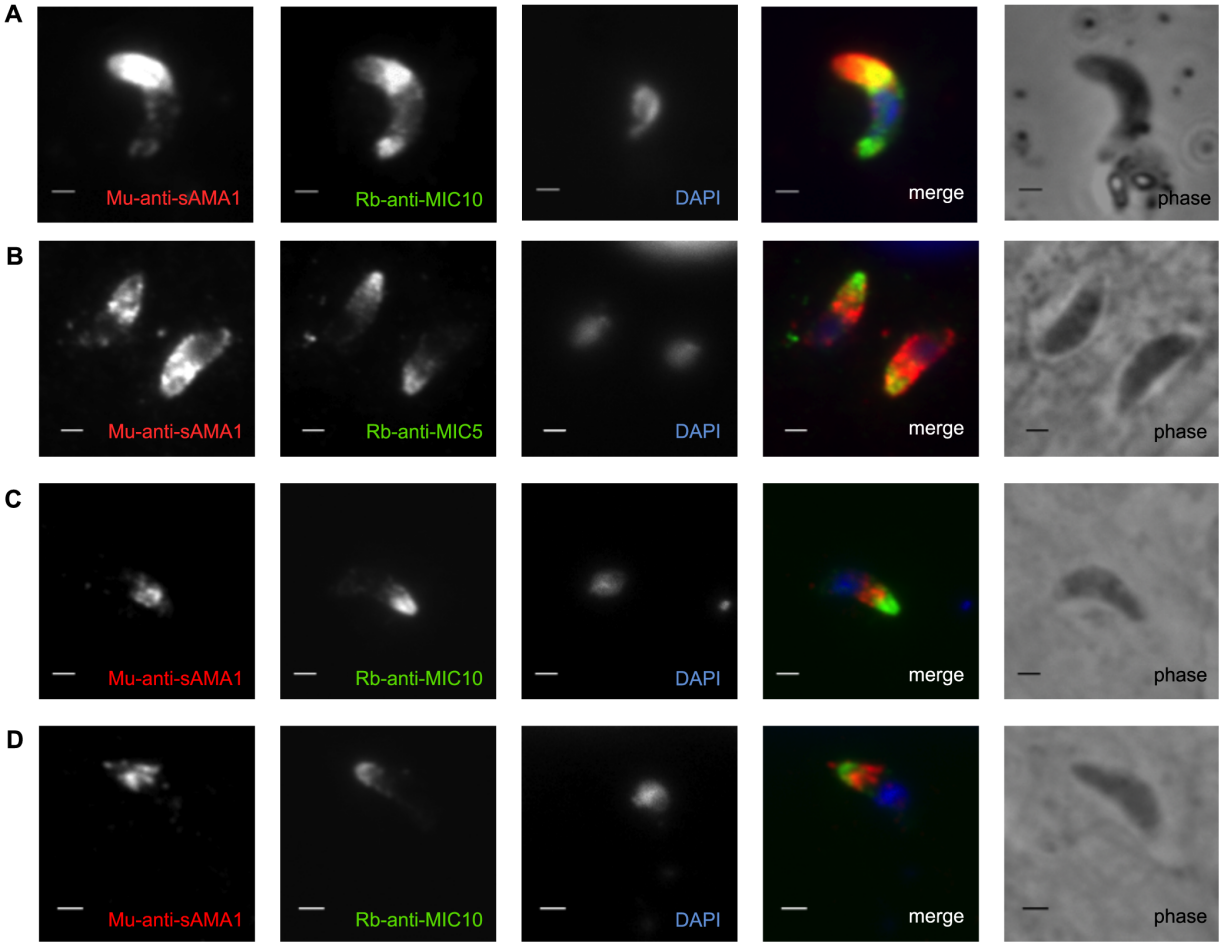
SporoAMA1 localizes apically in sporozoites. Extracellular sporozoites (A) or HFF monolayers infected with sporozoites for 2–3 hours (B–D), were formaldehyde-fixed, permeabilized with triton X-100, and stained with mouse anti-sporoAMA1 (Mu-anti-sAMA1) and rabbit (Rb) anti-MIC5 (B) or anti-MIC10 (A, C and D). Images shown in (C) and (D) are for adjacent parasites in the same field that were too far apart to capture in one image; both are shown to convey the reproducibility of the pattern observed. Scale bars represent 2 µm.

In tachyzoites, it has been shown that generic AMA1 resides in the micronemes [3] and generic RON2 is localized in rhoptry necks [18], [31]. To determine the approximate localization of sporoAMA1 in the sporozoite stage, we raised antibodies to the sporoAMA1 ectodomain (amino acids 79-569) in mice. Extracellular sporozoites were plated on glass slides, fixed, permeabilized and stained with antibodies to MIC10 and sporoAMA1 (Fig. 5A). In extracellular sporozoites, sporoAMA1 localizes to the apical end of the parasite, similar to the pattern seen with MIC10 and similar to what has been observed in tachyzoites [32], [33], [34]. Attempts to stain unpermeabilized sporozoites gave highly variable patterns that were not considered reliable (data not shown). We then examined sporoAMA1 localization in intracellular sporozoites. Freshly invaded sporozoites were fixed and permeabilized followed by incubation with anti-sporoAMA1 polyclonal sera and with anti-MIC10 and anti-MIC5, another protein that has been localized previously to the micronemes of tachyzoites [32], [33], although like MIC10, it has not been studied in sporozoites. Although all three markers showed consistent apical staining, no significant co-localization was observed between sporoAMA1 and either of the other two markers, with sporoAMA1 localizing distally to the extreme apical location of the two MIC markers.

**Figure 6:**
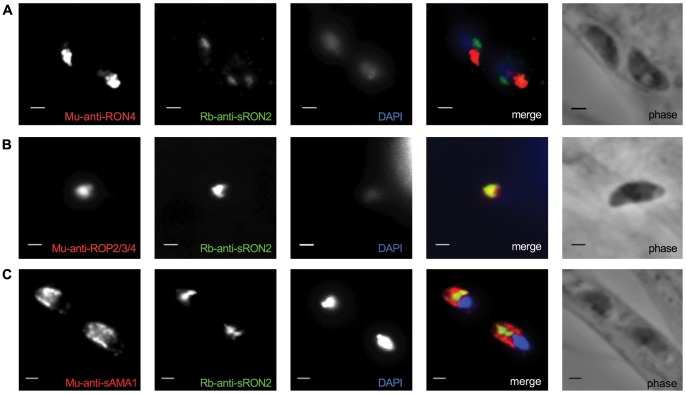
SporoRON2 shows partial colocalization with ROP2/3/4 but little if any with RON4. Infected HFF monolayers were infected with M4 sporozoites for 2–3 hours, and then were methanol-fixed, and stained with rabbit anti-sporoRON2 (Rb-anti-sRON2) and either mouse (Mu) anti-RON4 (A), anti-ROP2/3/4 (B), or anti-sporoAMA1 (C). Images where two parasites were present in the same field are shown except for (B) where no such fields were found. The image shown in (B), however, is representative of the pattern consistently observed with these two antibodies. Scale bars represent 2 µm.

To determine localization of sporoRON2, we raised rabbit antibodies to domain 4 (amino acids 1069-1167) of sporoRON2 that was fused to GST. Freshly invaded sporozoites were fixed in methanol and stained with anti-sporoRON2 polyclonal sera. The results were compared with the localization of either RON4 (a Rhoptry Neck protein detected in tachyzoites and the proteome of sporozoites) (Fig. 6a) or ROP2/3/4 (Rhoptry Bulb proteins also abundantly detected in proteomes of both stages) (Fig. 6b) [31]. Surprisingly, sporoRON2 exhibited no colocalization with RON4 but partial colocalization with ROP2/3/4. No colocalization was found between sporoAMA1 and sporoRON2 (Fig. 6c). These results suggest the possibility that sporoRON2 may localize, in part, to the rhoptry bulbs. Although we cannot exclude the possibility that the antibody is detecting an unrelated sporozoite protein, they were raised to a relatively short portion of sporoRON2 (∼100 amino acids) that has no significant similarity to any other predicted protein in *Toxoplasma* and the antibodies were shown to not react to generic RON2 (or any other protein) in tachyzoites (data not shown). Unfortunately, we were not able to obtain enough sporozoites for studies by immuno-electron microscopy, and there are no well-studied markers for sporozoite organelles. Hence, we can draw no definitive conclusions about the exact localization of any of these proteins but the results seen suggest substantial differences from the paradigms developed with generic AMA1 and RON2 in tachyzoites. Note that despite several attempts, we were unable to identify sporozoites that were unambiguously in the process of invasion and so cannot comment on which, if either, of the AMA1/RON2 pairs described here is at the MJ formed by invading sporozoites.

### Treatment of Sporozoites with sporoRON2-D3 Impedes Sporozoites Invasion

**Figure 7:**
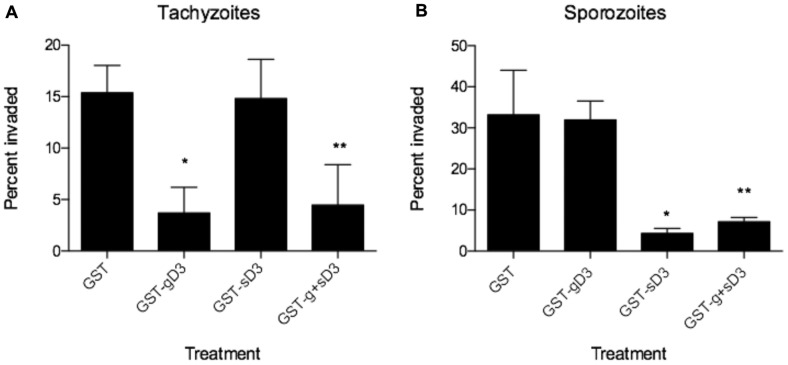
Preincubation of parasites with GST-sporoRON2-D3 specifically impedes sporozoite but not tachyzoite invasion. Tachyzoites (A) and sporozoites (B) were pretreated with molar equivalents of GST, GST-gD3, GST-sD3, and a mixture of GST-gD3/GST-sD3, and then permitted to invade a monolayer of HFFs for 45 minutes, following temperature synchronization. The number of intracellular parasites was determined by differential staining before and after permeabilization. The percent of invaded (intracellular) parasites relative to the total number was determined by counting parasites in 10 randomly selected fields from each of three coverslips for each condition. The counting and analysis were done blinded. A single asterisk indicates p<0.05 for the difference relative to the GST control; double asterisks indicate p<0.01 relative to this control.

Blocking the generic AMA1/RON2 interaction by incubating tachyzoites in *Toxoplasma* or merozoites in *Plasmodium* with generic RON2 domain 3 inhibits invasion [17], [18], [35]. To test whether the generic AMA1/RON2 complex and/or the sporozoite AMA1/RON2 complex play a role in sporozoite invasion, we performed invasion-inhibition assays of sporozoites and tachyzoites in the presence of equimolar amounts of either GST-gD3, GST-sD3, both or GST alone. Extracellular vs. intracellular parasites were identified by sequential staining with polyclonal rabbit anti-*Toxoplasma* antisera before detergent permeabilization of host cells, and with mouse anti-SAG1 sera after permeabilization. This allowed us to discriminate successful from blocked invasion events. The results (Fig. 7a) showed that treatment of tachyzoites with GST-gD3 significantly decreases the efficiency of invasion, as previously reported [18], while GST-sD3 has no such effect, as predicted from our biochemical and structural studies.

Interestingly, even though the generic AMA1/RON2 pair is expressed in sporozoites, treatment with GST-gD3 had no effect on sporozoite invasion. Treatment with GST-sD3, however, resulted in a significant decrease (∼88%) in the invasion rate (Fig. 7b). Sporozoites treated with both GST-gD3 and GST-sD3 were also markedly decreased in their invasion efficiency although to a slightly lesser degree (∼70%), likely due to the fact that there was only half as much GST-sD3 in that fraction compared to the GST-sD3 alone (to keep total protein added equal). These results indicate that the interaction of sporoAMA1 and sporoRON2-D3 is important for successful invasion of host cells by sporozoites, while the interaction of generic AMA1/RON2 appears dispensable, at least in these conditions.

## Discussion

The results presented here demonstrate that invasion of *Toxoplasma* sporozoites depends on the interaction of two previously uncharacterized proteins, sporoRON2 and sporoAMA1. These proteins form a specific pairing, similar to the previously described interaction of generic AMA1 and generic RON2 [13], [18]. This finding begs the question as to why the sporozoites evolved to use a novel pairing rather than the generic counterparts. Homologues of sporoRON2/sporoAMA1 are found in *Neospora* and *Eimeria*, but are missing in *Theileria* and *Plasmodium* species. Unlike their *Haemospororina* relatives, which are insect-transmitted, sporozoites in the *Eimeriorina* suborder have to cross the intestinal epithelium of their intermediate hosts to initiate a new infection. SporoRON2 and sporoAMA1, therefore, may represent an effective solution to that unique challenge. The mammalian gut presents a formidable barrier to invading microorganisms with defenses ranging from the unique morphology of enterocytes (extensive microvilli and a robust cytoskeleton) to the presence of abundant mucus and a somewhat alkaline pH [36], [37]. It has been previously reported that *Toxoplasma* sporozoites may cross the intestinal epithelial cells before replicating in the cells of lamina propria [38], [39], and it may be that the sporoAMA1/sporoRON2 pairing allows the sporozoites of *Eimeriorina* species to cross this barrier more efficiently than the generic AMA1/RON2 pair. If crossing the intestinal epithelium is the major explanation for the presence of sporoAMA1/sporoRON2, one might expect *Toxoplasma* bradyzoites, which are also orally infectious, to deploy this pairing. Based on transcriptomic analyses, however, bradyzoites do not appear to express either of these genes [24].

Our data do not address why generic versions of AMA1/RON2 are also present in sporozoites. It has been previously reported that, upon invasion, sporozoites form an unusually large primary vacuole, in which the parasite remains for the first 12–18 hours [22], [40]. Following this, the parasite creates another moving junction and forms a secondary vacuole, in which it then replicates (note that while we did not observe any particularly spacious (i.e. primary) vacuoles, we did not follow invasion by video microscopy and so may have missed this two-step process if under our conditions primary vacuoles are not readily distinguishable from secondary vacuoles). Such a two-step process could be another way in which the two AMA1/RON2 pairings operate: e.g., the sporozoite-specific pairing might play a role in the first step (as argued by our invasion inhibition studies) while the generic pair operates in the more tachyzoite-like formation of the secondary vacuole. Finally, it might also be that the generic and sporozoite-specific AMA1/RON2 pairs are secreted under different invasion conditions; this possibility cannot be excluded as we have looked only *in vitro* and only with fibroblasts as the target host cell.

**Figure 8:**
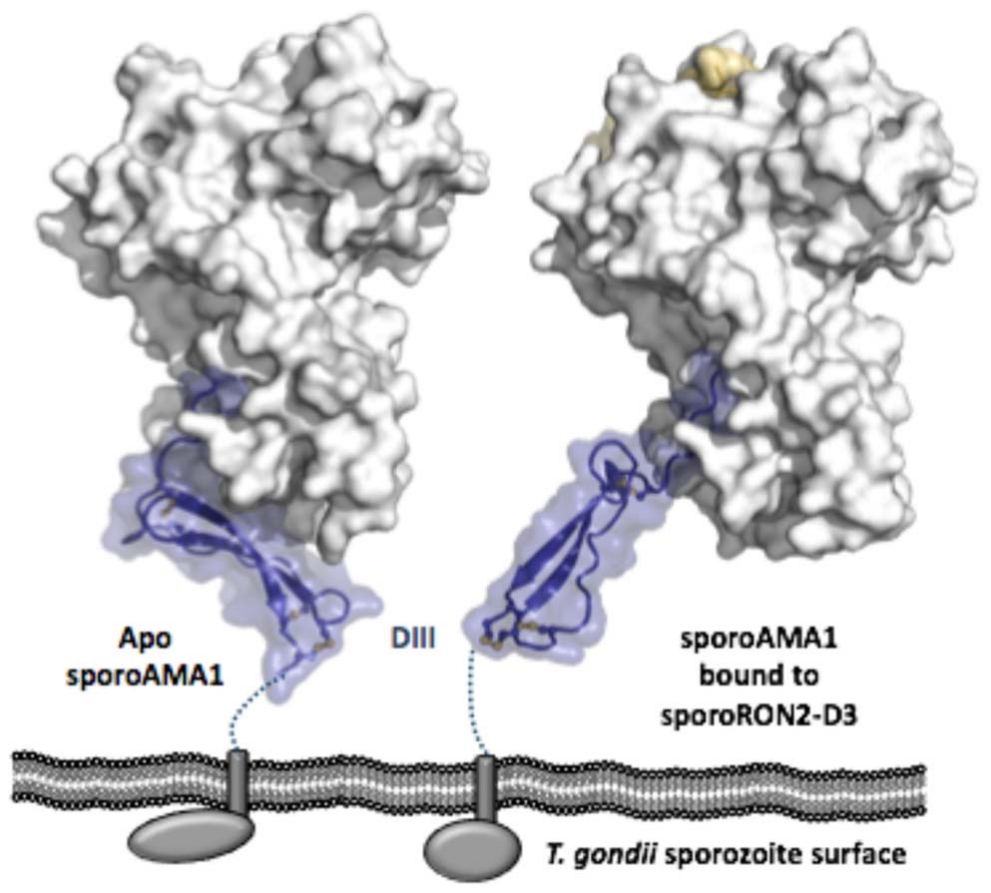
SporoAMA1 DIII reorganization upon ligand binding provides possible insight into signal transduction mechanisms. SporoAMA1 shown in predicted organization to the *T. gondii* sporozoite plasma membrane, with DI and DII shown as a grey surface and DIII shown as blue cartoon with a semi-transparent blue surface, sporoRON2-D3 shown as a gold surface, and disulfides shown as yellow sticks. Dotted lines indicate extended Pro/Glu rich region between the conserved portion of DIII and the transmembrane domain (grey rectangle) that leads through to the C-terminal domain (grey oval/sphere). Left – Apo SporoAMA1. Right – SporoAMA1 bound to sporoRON2-D3.

Previous studies have also postulated that the generic AMA1/RON2 interaction is involved in a signaling event that provides information to the intracellular environment upon formation of the moving junction complex [13], [26], [41]. While AMA1 domains I and II are both involved in coordinating RON2, and a significant conformational change occurs with the displacement of the domain II loop, this signal would still have to be passed through domain III in order to reach the cell interior. A possible mechanism for this signaling is observed when the domain III positioning of apo and bound sporoAMA1 is compared (Fig. 8). In the apo structure, the highly compact cysteine-knot containing DIII of sporoAMA1 extends across the base of domains I and II (Fig. 8 left), similar to the conformations observed for domain III in the generic AMA1 of *Toxoplasma* and of *Neospora* and *Babesia* [25], [26]. However, in the sporoAMA1-sporoRON2-D3 co-structure reported here, sporoAMA1 domain III is rotated away from the domain I/domain II core by about 90°, displacing the C-terminus by more than 40 Å (Fig. 8 right). It is noteworthy that only the compact three-domain organization is observed in the structural studies of generic AMA1. It is tempting to speculate that this conformational change at the base of sporoAMA1 is tied to the role of domain III as the conduit for a signal moving from the ectoplasmic region of AMA1 through to the intracellular domain. While a signaling role mediated by an articulating domain III is speculative, our structural data clearly demonstrates the inherent ability of domain III to adopt structurally distinct conformations. It is also worth noting that domain III of sporoAMA1 is the least conserved portion of the protein relative to its generic paralogue with an extended pro-glu linker between the cysteine knot and the transmembrane domain (displayed as a dashed line in Fig. 8). Since this linker is not included in the structurally characterized sporoAMA1, it is possible that the complete ectodomain tethered to the parasite cell surface might preferentially stabilize one of the two DIII orientations observed, independent of sporoRON2-D3.

Our attempts to localize sporoAMA1 and sporoRON2 in sporozoites showed the expected apical staining but surprising differences with other micronemal and rhoptry markers. Sporozoites have not been well studied because of the difficulty of working with them and so no well described, “gold standards” exist for the various organelles. It is known, however, that sporozoites have far more micronemes than tachyzoites and it could be that there are different subpopulations of micronemes with distinct cargoes in the sporozoites. The lack of colocalization between sporoRON2 and RON4 is more surprising and not easily explained. There is no *a priori* reason why a homologue of RON2, which in tachyzoites colocalizes with RON4 in the rhoptry necks, must also go to this compartment in the sporozoites. The localization data suggest that sporoRON2 might function independently of RON4 and, again, there is no *a priori* reason why this should not be the case. As mentioned above, the presumptive signaling that occurs when sporoRON2 binds sporoAMA1 could well operate independent of RON4 whose interaction with RON2 and overall role in the MJ complex has not been elucidated.

Taken together, our data provide new insight into the AMA1/RON2 interaction and show that evolution has favored a structurally distinct pairing in the form of sporoRON2/sporoAMA1 in *Toxoplasma* sporozoites and, we presume, sporozoites of the other Eimeriorina where orthologues for these genes are observed. This provides further evidence that this interaction is critical for Apicomplexan invasion of host cells although the enigma remains of why the generic AMA1/RON2 pair is expressed in sporozoites if not necessary for their invasion.

## Materials and Methods

### Host Cell Culture and Parasites

Human foreskin fibroblasts (HFFs) were cultured in complete Dulbecco’s modified Eagle’s medium (DMEM; Invitrogen, Carlsbad, CA) supplemented with 10% heat-inactivated fetal calf serum (Hyclone, Logan, UT), 2 mM L-glutamine, 100 U ml^−1^ penicillin and 100 µg ml^−1^ streptomycin. *Toxoplasma gondii* (RH and ME49 strains) lacking a functional hypoxanthine-xanthine-guanine phosphoribosyltransferase (*HXGPRT*) gene (designated RHΔ*hxgprt* or ME49Δ*hxgprt,* respectively) [42] were cultured by serial passage on confluent monolayers of HFFs in complete DMEM at 37°C with 5% CO2.

### Generation of GST-RON2 (gD3 and sD3) Fusion Proteins

To generate RON2 fusion proteins with a N-terminal GST tag, genericRON2 and sporoRON2 sequences were aligned to determine the location of domain 3 (D3) of sporoRON2. Once determined, both generic RON2*-*D3 and sporoRON2*-*D3 were PCR-amplified from *Toxoplasma* ME49 genomic DNA and introduced into pGEX-6P1 (Agilent) using the BamHI and EcoRI sites. GST-gD3 was generated as described previously [18]. To generate GST-sD3, the coding sequence for Tg sporoRON2 amino acids 995 to 1048 was PCR amplified using primers (GGATCCGACATCGCTCAGTTCCTCACCGAC and CTCGAGTCACTTGAAGACATCCGACAGCGCAG). Production and purification of the GST proteins from *E. coli* strain Rosetta (Novagen) were done essentially as described previously [43]. Concentrated, purified proteins were stored at −80°C in buffer containing 10 mM Tris-HCl pH 8.0, 150 mM NaCl, and 10% glycerol.

### Generation of sporoAMA1-HA Plasmid

RNA was extracted from sporozoites using Trizol reagent as previously described [24], cDNA was generated using random hexamer primer and the sporoAMA1 gene was amplified using the following primers: sporoAMA1-FW (ATGCCTACAGAATCTCGAAGTAT) and sporoAMA1-REV (GAACTCTGCGTCGACGGCCCT). The resulting product was then subcloned into TOPO for sequencing. After verifying the correct sequence the gene was cloned using the cold-fusion kit (Systems Biosciences, Mountain View, USA), into pSAG1-CDPK3::HA [44] using the primers : sporoAMA1_cold-FW (CGAGTATGcatgccATGCCTACAGAATCTCGAAGTATCTTGGCTAGGGCGGAAGAGACC) and sporoAMA1_cold-REV: (CAACGGTGAttaATTAATCAGAACTCTGCGTCGACGGCCCTGGAACCCAGAAGCGACT) to generate pSAG1-sporoAMA1::HA.

### Transfection of RHΔ*hxgprt* Parasites

2×10^7^ RHΔ*hxgprt* tachyzoites were washed once with 1×PBS. After 1 wash in Cytomix (10 mM KPO_4_, 120 mM KCl, 0.15 mM CaCl_2_, 5 mM MgCl_2_, 25 mM Hepes, 2 mM EDTA), the parasites were transfected with 50 µg of pSAG1-sporoAMA1::HA as previously published [45]. Parasites were plated on a fully confluent HFF monolayer, allowed to invade for 24 hours before the media was changed to DMEM to remove residual cytomix.

### GST Pull-down Experiments

Approximately 4×10^8^ extracellular RHΔ*hxgprt* or RHΔ*hxgprt-*sporoAMA1-HA parasites were washed three times in 1× phosphate-buffered saline (PBS) and then lysed on ice in 1 ml of lysis buffer (10 mM Tris-HCl pH 8.0, 150 mM NaCl, 1 mM EDTA, 0.1% NP-40), supplemented with Complete EDTA-free Protease Inhibitors (Roche). The cleared, NP-40-solublized lysate was divided equally into three tubes and each fraction was supplemented with 4B Glutathione-sepharose beads (GE Healthsciences) that were prebound with 0.5 µM of GST, GST-gD3, or GST-sD3. The lysate suspensions were rotated at room temperature for approximately two hours and the bound beads were then centrifuged at 70 g for 2 minutes and the supernatant (“flow-through”) collected. The pelleted beads were then washed three times in lysis buffer, followed by elution of the GST fusion proteins and any copurified parasite proteins by boiling for ∼5 minutes in 2× SDS sample buffer (125 mM Tris-HCl pH 7.0, 4% SDS, 20% glycerol, 0.005% bromophenol blue) supplemented with 10% β-mercaptoethanol (“pull-down”).

### Western Blot Analyses

Samples were separated on 4–12% gradient Bis-Tris gels (Invitrogen) and analyzed by Western Blot using the following antibodies. Generic AMA1 was detected with mouse monoclonal B3.90 [46]. SporoAMA1-HA was detected using the anti-HA rat monoclonal antibody 3F10 conjugated to horseradish peroxidase (HRP) (Roche). SAG1 was detected using rabbit polyclonal sera (a gift from M. Grigg, NIH). Goat anti-mouse secondary antibodies were HRP-conjugated (Biorad).

### Protein Production

A sequence encoding the fully processed ectoplasmic region of sporoAMA1 (Ser79 to Asn569, with numbering based on the initiation methioine; sporoAMA1full) was synthesized by GenScript and codon optimized for insect cells. A construct encompassing just the conserved portions of the three ectoplasmic domains was sub-cloned out of the synthesized gene (Gln98 to Glu481; sporoAMA1c) into a modified pAcGP67B vector (Pharmigen) incorporating a C-terminal hexahistidine tag separated from sporoAMA1 by a thrombin cleavage site. SporoAMA1-encoding viruses for insect cell protein production were generated and amplified using established protocols [13], [25]. For large scale expression, baculovirus infected Hi-5 cells were incubated for 65 hours, following which the supernatant was harvested, concentrated, buffer-exchanged, and allowed to batch-bind with Ni-agarose beads at 4°C for 1 hour. SporoAMA1 constructs were eluted with buffer containing 250 mM imidazole, and fractions were analyzed by SDS-PAGE and pooled based on purity. The His_6_ tag was removed by thrombin cleavage, and sporoAMA1 was further purified by size exclusion chromatography (Superdex 16/60 200) in HEPES buffered saline (HBS). Both constructs were concentrated to 30 mg/mL for crystallization trials, while sporoAMA1c was also used for co-purification with sporoRON2-D3.

For domain 3 of sporoRON2, the sequence encoding the region of sporoRON2 corresponding to the generic RON2 synthetic peptide [13] except for the exclusion of two hydrophobic residues at the C-terminus of the sporoRON2 region (Asp999 to Ser1034 with numbering based on the initiation methionine), was synthesized by GenScript, codon-optimized for expression in *Escherichia coli*, and cloned into a modified pET32a vector (Novagen) containing N-terminal thioredoxin (TRX) and His_6_ tags separated from the gene of interest by a thrombin site. The sporoRON2-D3-TRX fusion was produced recombinantly in *E. coli* BL21 codon plus (DE3) cells (Novagen) grown at 30°C in autoinduction medium (Novagen). Following 16 hrs of growth, the cell pellet was harvested by centrifugation, resuspended in buffer and frozen at −80°C.

### Protein Co-purification

To prevent aggregation, sporoRON2-D3-TRX was purified in complex with sporoAMA1c. The sporoRON2-D3-TRX sample was thawed on ice, lysed in a French Press, and insoluble material was removed by centrifugation. Purified, cleaved sporoAMA1c was added directly to the sporoRON2-D3-TRX clarified cell lysate and allowed to incubate at 4°C for 30 min. sporoRON2-D3-TRX and any bound sporoAMA1c was purified by Ni batch binds as described above for sporoAMA1c. The TRX tag was removed by overnight thrombin cleavage, and the sporoAMA1c-sporoRON2-D3 complex was purified from thrombin, thioredoxin tag, excess sporoRON2-D3 and other contaminating proteins by size exclusion chromatography. The purified sample was concentrated to 30 mg/mL in HBS for crystallization trials. The purity of the complex was determined by SDS-PAGE at each stage of the purification and protein concentrations were analyzed by absorbance at 280 nm.

### Crystallization, Data Collection and Processing

#### sporoAMA1

While sporoAMA1full proved refractory to crystallization, initial crystals of sporoAMA1c were identified in MCSG-1 (Microlytic) after 12 days. The optimized crystals grew to their final size within 8 weeks in a condition of 1.0 M succinic acid pH 7.0, 100 mM HEPES pH 7.0, 1% PEG monomethyl ether 2000, and 100 mM glycine. The final 2.4 µL drops consisted of equal volumes sporoAMA1c (30 mg/mL) and reservoir solution equilibrated against 100 µL of reservoir solution. Cryoprotection was carried out in 25% glycerol for 20 seconds and the crystal was flash cooled at 100K directly in the cryostream. Diffraction data to 2.35 Å resolution were collected on beam line 9-2 at the Stanford Synchrotron Radiation Lightsource (SSRL; Menlo Park, CA).

#### sporoAMA1c-sporoRON2-D3

Crystal trials for sporoAMA1c-*Tg*sporoRON2-D3 were set using a Crystal Gryphon (Art Robbins Instruments) crystallization robot using several different commercial crystallization screens and three different protein to reservoir drop ratios. Initial crystals of sporoAMA1c-sporoRON2-D3 were identified in Index (Hampton Research), and diffraction quality crystals were obtained after two days in 0.2 M magnesium chloride hexahydrate, 0.1 M HEPES pH 7.5, 25% PEG3350. The final 0.6 µL drops consisted of 0.2 µL protein (sporoAMA1c and sporoRON2-D3 (15 mg/mL combined)) and 0.4 µL reservoir solution equilibrated against 50 µL of reservoir solution. Cryoprotection was carried out in 25% glycerol for 20 seconds and the crystal was flash cooled at 100K directly in the cryostream. Diffraction data to 3.10 Å resolution were collected on the micro-focus beam line 12-2 at SSRL.

### Structure Solution and Refinement

Diffraction data were processed using Imosflm [47] and Scala [48] in the CCP4 suite of programs [49]. Initial phases were obtained by molecular replacement (MR) using PHASER [50] **.** For sporoAMA1c, the MR model consisted of the DI and DII domains of the unliganded generic AMA1 structure (PDB ID 2X2Z) trimmed with CHAINSAW [51] to better reflect the sporoAMA1 sequence. No MR solution was found for DIII, which was manually traced into the electron density after several rounds of refinement of the core structure. For sporoAMA1c*-*sporoRON2-D3, the MR model consisted of the DI and DII domains of the unliganded sporoAMA1c structure with the DII loop removed. No MR solution was found for DIII, and was manually traced into the electron density after multiple rounds of refinement. Tracing of sporoRON2-D3 and addition of solvent molecules, was performed manually in COOT [52]. Addition of solvent molecules was performed manually in COOT [52] and refinement in Refmac5 [53]. Stereochemical analysis performed for each structure with PROCHECK and SFCHECK in CCP4 [49] showed good stereochemistry with more than 95% of the residues in the favored conformations and no residues modeled in disallowed orientations of the Ramachandran plot. Overall 5% of the reflections were set aside for calculation of sporoAMA1c R_free_ while 10% were set aside for sporoAMA1c-sporoRON2-D3 R_free_. Data collection and refinement statistics are presented in Table 2.

**Table 2:** Data collection and refinement statistics.

	sporoAMA1c apo	sporoAMA1c-sporoRON2-D3
***Data collection***		
Spacegroup	P2_1_	C222_1_
a, b, c (Å)	179.19, 155.52, 180.59	49.37, 124.18, 171.91
α, β, γ (deg.)	90, 92.31, 90	90, 90, 90
Wavelength (Å)	0.9795	0.9795
Resolution range (Å)	79.30–2.35 (2.48–2.35)	85.96–3.10 (3.27–3.10)
Measured reflections	1391080	39279
Unique reflections	391285	9716
Redundancy	3.6 (2.4)	4.0 (3.7)
Completeness (%)	95.4 (75.8)	97.8 (95.8)
*I/σ(I)*	10.0 (2.1)	8.2 (3.5)
R_merge_ a	0.081 (0.341)	0.114 (0.311)
***Refinement Statistics***		
Resolution (Å)	78.04–2.35 (2.41–2.35)	85.96–3.10 (3.18–3.10)
R_work_ b	0.209 (0.331)	0.205 (0.311)
R_free_ c	0.244 (0.353)	0.263 (0.378)
No. of atoms		
Protein (A/B/C/D/	2927/2899/2899/2897	2745/229
E/F/G/H/	2927/2889/2927/2913/	
I/J/K/L)	2865/2891/2921/2921	
Solvent	1695	7
Glycerol	108	N/A
B-values (Å^2^)		
Protein (A/B/C/D/E/	37.5/35.7/36.3/35.6/36.0/	49.5/58.6
F/G/H/I/J/	38.2/38.2/38.7/46.1/48.3/	
K/L)	49.9/50.0	
Solvent	43.7	31.7
Glycerol	43.6	N/A
r.m.s. deviation from ideality		
Bond lengths (Å)	0.013	0.011
Bond angles (deg.)	1.31	1.22
Ramachandran statistics (%)		
Most favoured	96.1	95.9
Allowed	3.9	4.1
Disallowed	0.0	0.0

Values in parentheses are for the highest resolution shell.

a R_merge_ = ∑*_hkl_* ∑*_i_* |I*_hkl,i_* - [I*_hkl_*]|/∑*_hkl_* ∑*_i_* I*_hkl,i_*, where [I*_hkl_*] is the is the average of symmetry related observations of a unique reflection.

b R_work_ = ∑|F_obs_-F_calc_|/∑F_obs_, where F_obs_ and F_calc_ are the observed and the calculated structure factors, respectively.

c R_free_ is R using 5% (apo) or 10% (complex) of reflections randomly chosen and omitted from refinement.

### Protein Data Bank Accession Codes

The atomic coordinates and structure factors have been deposited in the Protein Data Bank under the following codes: sporoAMA1c – PDB ID: 3ZLE, r3ZLEsf; sporoAMA1c in complex with sporoRON2-D3– PDB ID: 3ZLD, r3ZLDsf.

### Generation of anti-Tg sporoAMA1 Ectodomain Antibodies

Antibodies were raised against the sporoAMA1 ectodomain that was purified as stated above. The ectodomain was dissolved in PBS and 100 µg was injected into BALB/c mice in RIBI adjuvant (Corixa). Identical boosts were given at 21-day intervals. Polyclonal antisera was collected after the second boost and screened for reactivity by immunofluorescence analysis (IFA) against tachyzoites transfected with sporoAMA1-HA. Antibodies used in this study showed colocalization with anti-HA parasites expressing sporoAMA1-HA but no reactivity with untransfected parasites indicating their specificity for sporoAMA1.

### Generation of anti-Tg sporoRON2 Domain 4 Antibodies

Antibodies were raised to GST fusions of Domain 4 of sporoRON2 (amino acids 1069 = 1167). Proteins were purified as described above (GST purification). 500 ug was injected subcutaneously into rabbits (New Zealand White, Harlan laboratories). A 1∶1 mix with Freund's complete adjuvant was used for the first injection only. Freund's incomplete was used for all subsequent boosts. Boosts were done every 2 weeks until a sufficiently high titer was obtained. Polyclonal antisera was collected after the 3rd injection and at each subsequent boost. Bleeds were screened for reactivity to sporozoites (by immunofluorescence), and showed no reactivity to tachyzoites (as determined by Western blot and immunofluorescence).

### Sporozoite Excystation

Oocysts were produced in kittens and harvested from feces as previously described [24]. Sporulated M4 oocysts that had been stored in 2% sulfuric acid at 4°C were washed three times in 1× PBS to remove sulfuric acid. The final washed pellet was suspended in 10% Clorox® bleach (diluted in 1× PBS) and incubated on ice for 30 minutes. Oocysts were then washed two times with 1× PBS and a third time in DMEM media (without serum) to remove bleach. The final washed oocyst pellet was suspended in DMEM and transferred to a 1.5 ml screw-top microcentrifuge tube containing 350 mg acid-washed glass beads (200–400 mm, Invitrogen) and vortexed at max speed in three 30-second intervals (90 seconds total) at which time approximately 90% of the oocysts were broken open with free sporocysts. Broken oocysts/sporocysts were collected and pelleted by centrifugation. The resulting pellet was suspended in 5% sodium taurodeoxycholate hydrate (Sigma) in DMEM and incubated at 37°C for 10 minutes. Sporozoites were then washed two times in cold DMEM. A third wash was performed in DMEM supplemented with 2% FBS. The final washed sporozoites were suspended in DMEM supplemented with 2% FBS for invasion assay.

### Immunofluorescence Assays

To visualize Tg sporoAMA1 in extracellular sporozoites, sporozoites were excysted and placed on a 12-well glass slide (Tekdon, Inc.). The sporozoites were allowed to dry on the slide before being fixed in 1× PBS with 2.5% formaldehyde (EM Biosciences) and permeabilized using 0.2% Triton X-100 in PBS and 3% Bovine Serum Albumin (Sigma). To visualize sporoAMA1 in invaded sporozoites, HFF monolayers infected with sporozoites were fixed in 1× PBS containing 2.5% formaldehyde (EM Biosciences), 2–3 hours post-infection followed by permeabilization as for extracellular parasites. The monolayers were stained with polyclonal mouse-anti-sporoAMA1 serum, and either rabbit anti-MIC10 or rabbit-anti-MIC5 polyclonal sera. The primary staining was followed by AlexaFluor594-goat-anti-mouse antibody and AlexaFluor488-goat-anti-rabbit antibody, respectively. All secondary AlexaFluor-conjugated antibodies were obtained from Molecular Probes. Coverslips were mounted onto glass slides using Vectashield (Vector Laboratories) and then examined using 100× oil-immersion lens on an Olympus BX60 upright fluorescent microscope.

To visualize sporoRON2 in apical compartment of sporozoites, HFF monolayers infected with sporozoites were fixed in 100% ice-cold methanol for 3 minutes at room temperature. These fixed monolayers were then blocked in PBS and 3% Bovine Serum Albumin (Sigma). The monolayers were stained with polyclonal rabbit-anti-sporoRON2 serum and either mouse-anti-RON4, mouse-anti-ROP2/3/4 or mouse-anti-sporoAMA1. The primary staining was followed by AlexaFluor594-goat-anti-mouse antibody and AlexaFluor488-goat-anti-rabbit antibody, respectively (Invitrogen). Coverslips were mounted onto glass slides using Vectashield (Vector Laboratories) and then examined using 100× oil-immersion lens on an Olympus BX60 upright fluorescent microscope.

All digital images were obtained using Image-Pro Plus and the same exposure parameters were used for all comparison sets.

### Invasion Assay

ME49 tachyzoites were released from infected HFFs by scraping and passage through a 27 gauge needle. Released tachyzoites and M4 excysted sporozoites (as described above) were washed in DMEM supplemented with 2% FBS. Parasites were then incubated in DMEM with 2% FBS supplemented with: 5 µM GST, 5 µM GST-gD3, 5 µM GST-sD3, or 2.5 µM of both GST-gD3 and GST-sD3 at 37C for 3 minutes. (All proteins were in 10 mM Tris-HCl pH 8.0, 150 mM NaCl, 10% glycerol.) Parasites were added to pre-chilled HFF monolayers grown on glass coverslips. Temperature synchronization of invasion was accomplished by allowing parasites to settle onto the HFFs in an ice water bath for 10 minutes prior to invasion. To initiate invasion, the plates were then transferred to a 37°C water bath for 45 minutes. Infected monolayers were washed twice in 1× PBS and then fixed in 1× PBS containing 2.5% formaldehyde. To stain only the extracellular parasites, fixed monolayers were stained with polyclonal rabbit-anti-*Toxoplasma* serum followed by AlexaFluor488-goat-anti-rabbit. To stain all parasites, the infected monolayers were then permeabilized with 1× PBS containing 0.2% triton X-100 followed by staining with the anti-SAG1 mouse monoclonal antibody DG52 [54] and AlexaFluor594-goat-anti-mouse. The numbers of green (extracellular) and red (intracellular and extracellular) tachyzoites were counted in 10 randomly selected fields on each of three separately mounted coverslips for each condition and visualization was performed using a 20× lens on a Nikon Eclipse TE300 microscope. The same exact process was carried out for sporozoites. All image acquisition and analysis was done blinded. All digital images were obtained using Image-Pro Plus and parasites were quantified using ImageJ.

## References

[1] Dobrowolski JM, Sibley LD (1996) Toxoplasma invasion of mammalian cells is powered by the actin cytoskeleton of the parasite. Cell 84: 933–939. 8601316 10.1016/s0092-8674(00)81071-5

[2] Keeley A, Soldati D (2004) The glideosome: a molecular machine powering motility and host-cell invasion by Apicomplexa. Trends Cell Biol 14: 528–532. 15450974 10.1016/j.tcb.2004.08.002

[3] Alexander DL, Mital J, Ward GE, Bradley P, Boothroyd JC (2005) Identification of the Moving Junction Complex of Toxoplasma gondii: A Collaboration between Distinct Secretory Organelles. PLoS Pathog 1: e17. 16244709 10.1371/journal.ppat.0010017 PMC1262624

[4] Lebrun M, Michelin A, El Hajj H, Poncet J, Bradley PJ, et al (2005) The rhoptry neck protein RON4 re-localizes at the moving junction during Toxoplasma gondii invasion. Cell Microbiol 7: 1823–1833. 16309467 10.1111/j.1462-5822.2005.00646.x

[5] Alexander DL, Arastu-Kapur S, Dubremetz JF, Boothroyd JC (2006) Plasmodium falciparum AMA1 binds a rhoptry neck protein homologous to TgRON4, a component of the moving junction in Toxoplasma gondii. Eukaryot Cell 5: 1169–1173. 16835460 10.1128/EC.00040-06 PMC1489286

[6] Besteiro S, Michelin A, Poncet J, Dubremetz JF, Lebrun M (2009) Export of a Toxoplasma gondii rhoptry neck protein complex at the host cell membrane to form the moving junction during invasion. PLoS Pathog 5: e1000309. 19247437 10.1371/journal.ppat.1000309 PMC2642630

[7] Collins CR, Withers-Martinez C, Hackett F, Blackman MJ (2009) An inhibitory antibody blocks interactions between components of the malarial invasion machinery. PLoS Pathog 5: e1000273. 19165323 10.1371/journal.ppat.1000273 PMC2621342

[8] Straub KW, Cheng SJ, Sohn CS, Bradley PJ (2009) Novel components of the Apicomplexan moving junction reveal conserved and coccidia-restricted elements. Cell Microbiol 11: 590–603. 19134112 10.1111/j.1462-5822.2008.01276.x PMC2798130

[9] Richard D, MacRaild CA, Riglar DT, Chan JA, Foley M, et al (2010) Interaction between Plasmodium falciparum apical membrane antigen 1 and the rhoptry neck protein complex defines a key step in the erythrocyte invasion process of malaria parasites. J Biol Chem 285: 14815–14822. 20228060 10.1074/jbc.M109.080770 PMC2863225

[10] Aikawa M, Miller LH, Johnson J, Rabbege J (1978) Erythrocyte entry by malarial parasites: a moving junction between erythrocyte and parasite. J Cell Biol 77: 72–82. 96121 10.1083/jcb.77.1.72 PMC2110026

[11] Michel R, Schupp K, Raether W, Bierther FW (1980) Formation of a close junction during invasion of erythrocytes by Toxoplasma gondii in vitro. Int J Parasitol 10: 309–313. 7451022 10.1016/0020-7519(80)90012-0

[12] Suss-Toby E, Zimmerberg J, Ward GE (1996) Toxoplasma invasion: the parasitophorous vacuole is formed from host cell plasma membrane and pinches off via a fission pore. Proc Natl Acad Sci U S A 93: 8413–8418. 8710885 10.1073/pnas.93.16.8413 PMC38685

[13] Tonkin ML, Roques M, Lamarque MH, Pugniere M, Douguet D, et al (2011) Host cell invasion by apicomplexan parasites: insights from the co-structure of AMA1 with a RON2 peptide. Science 333: 463–467. 21778402 10.1126/science.1204988

[14] Tyler JS, Treeck M, Boothroyd JC (2011) Focus on the ringleader: the role of AMA1 in apicomplexan invasion and replication. Trends Parasitol 27: 410–420. 21659001 10.1016/j.pt.2011.04.002 PMC3159806

[15] Hehl AB, Lekutis C, Grigg ME, Bradley PJ, Dubremetz JF, et al (2000) Toxoplasma gondii homologue of plasmodium apical membrane antigen 1 is involved in invasion of host cells. Infect Immun 68: 7078–7086. 11083833 10.1128/iai.68.12.7078-7086.2000 PMC97818

[16] Mital J, Meissner M, Soldati D, Ward GE (2005) Conditional expression of Toxoplasma gondii apical membrane antigen-1 (TgAMA1) demonstrates that TgAMA1 plays a critical role in host cell invasion. Mol Biol Cell 16: 4341–4349. 16000372 10.1091/mbc.E05-04-0281 PMC1196342

[17] Lamarque M, Besteiro S, Papoin J, Roques M, Vulliez-Le Normand B, et al (2011) The RON2-AMA1 interaction is a critical step in moving junction-dependent invasion by apicomplexan parasites. PLoS Pathog 7: e1001276. 21347343 10.1371/journal.ppat.1001276 PMC3037350

[18] Tyler JS, Boothroyd JC (2011) The C-terminus of Toxoplasma RON2 provides the crucial link between AMA1 and the host-associated invasion complex. PLoS Pathog 7: e1001282. 21347354 10.1371/journal.ppat.1001282 PMC3037364

[19] Treeck M, Tamborrini M, Daubenberger CA, Gilberger TW, Voss TS (2009) Caught in action: mechanistic insights into antibody-mediated inhibition of Plasmodium merozoite invasion. Trends in parasitology 25: 494–497. 19734093 10.1016/j.pt.2009.07.008

[20] Pizarro JC, Vulliez-Le Normand B, Chesne-Seck ML, Collins CR, Withers-Martinez C, et al (2005) Crystal structure of the malaria vaccine candidate apical membrane antigen 1. Science 308: 408–411. 15731407 10.1126/science.1107449

[21] Dubey JP, Speer CA, Shen SK, Kwok OC, Blixt JA (1997) Oocyst-induced murine toxoplasmosis: life cycle, pathogenicity, and stage conversion in mice fed Toxoplasma gondii oocysts. J Parasitol 83: 870–882. 9379292

[22] Tilley M, Fichera ME, Jerome ME, Roos DS, White MW (1997) Toxoplasma gondii sporozoites form a transient parasitophorous vacuole that is impermeable and contains only a subset of dense-granule proteins. Infect Immun 65: 4598–4605. 9353039 10.1128/iai.65.11.4598-4605.1997 PMC175660

[23] Fritz HM, Bowyer PW, Bogyo M, Conrad PA, Boothroyd JC (2012) Proteomic analysis of fractionated Toxoplasma oocysts reveals clues to their environmental resistance. PLoS One 7: e29955. 22279555 10.1371/journal.pone.0029955 PMC3261165

[24] Fritz HM, Buchholz KR, Chen X, Durbin-Johnson B, Rocke DM, et al (2012) Transcriptomic analysis of toxoplasma development reveals many novel functions and structures specific to sporozoites and oocysts. PLoS One 7: e29998. 22347997 10.1371/journal.pone.0029998 PMC3278417

[25] Crawford J, Tonkin ML, Grujic O, Boulanger MJ (2010) Structural characterization of apical membrane antigen 1 (AMA1) from Toxoplasma gondii. J Biol Chem 285: 15644–15652. 20304917 10.1074/jbc.M109.092619 PMC2865318

[26] Tonkin ML, Crawford J, Lebrun ML, Boulanger MJ (2013) Babesia divergens and Neospora caninum apical membrane antigen 1 structures reveal selectivity and plasticity in apicomplexan parasite host cell invasion. Protein Sci 22: 114–127. 23169033 10.1002/pro.2193 PMC3575866

[27] Bai T, Becker M, Gupta A, Strike P, Murphy VJ, et al (2005) Structure of AMA1 from Plasmodium falciparum reveals a clustering of polymorphisms that surround a conserved hydrophobic pocket. Proc Natl Acad Sci U S A 102: 12736–12741. 16129835 10.1073/pnas.0501808102 PMC1200259

[28] Vulliez-Le Normand B, Tonkin ML, Lamarque MH, Langer S, Hoos S, et al (2012) Structural and functional insights into the malaria parasite moving junction complex. PLoS Pathog 8: e1002755. 22737069 10.1371/journal.ppat.1002755 PMC3380929

[29] Fauquenoy S, Morelle W, Hovasse A, Bednarczyk A, Slomianny C, et al (2008) Proteomics and glycomics analyses of N-glycosylated structures involved in Toxoplasma gondii–host cell interactions. Mol Cell Proteomics 7: 891–910. 18187410 10.1074/mcp.M700391-MCP200

[30] Krissinel E, Henrick K (2007) Inference of macromolecular assemblies from crystalline state. J Mol Biol 372: 774–797. 17681537 10.1016/j.jmb.2007.05.022

[31] Bradley PJ, Ward C, Cheng SJ, Alexander DL, Coller S, et al (2005) Proteomic analysis of rhoptry organelles reveals many novel constituents for host-parasite interactions in Toxoplasma gondii. J Biol Chem 280: 34245–34258. 16002398 10.1074/jbc.M504158200

[32] Brydges SD, Sherman GD, Nockemann S, Loyens A, Daubener W, et al (2000) Molecular characterization of TgMIC5, a proteolytically processed antigen secreted from the micronemes of Toxoplasma gondii. Mol Biochem Parasitol 111: 51–66. 11087916 10.1016/s0166-6851(00)00296-6

[33] Hoff EF, Cook SH, Sherman GD, Harper JM, Ferguson DJ, et al (2001) Toxoplasma gondii: molecular cloning and characterization of a novel 18-kDa secretory antigen, TgMIC10. Exp Parasitol 97: 77–88. 11281704 10.1006/expr.2000.4585

[34] Hehl A, Krieger T, Boothroyd JC (1997) Identification and characterization of SRS1, a Toxoplasma gondii surface antigen upstream of and related to SAG1. Mol Biochem Parasitol 89: 271–282. 9364971 10.1016/s0166-6851(97)00126-6

[35] Srinivasan P, Beatty WL, Diouf A, Herrera R, Ambroggio X, et al (2011) Binding of Plasmodium merozoite proteins RON2 and AMA1 triggers commitment to invasion. Proc Natl Acad Sci U S A 108: 13275–13280. 21788485 10.1073/pnas.1110303108 PMC3156155

[36] Hansson GC (2012) Role of mucus layers in gut infection and inflammation. Current opinion in microbiology 15: 57–62. 22177113 10.1016/j.mib.2011.11.002 PMC3716454

[37] Camilleri M, Madsen K, Spiller R, Greenwood-Van Meerveld B, Verne GN (2012) Intestinal barrier function in health and gastrointestinal disease. Neurogastroenterology and motility : the official journal of the European Gastrointestinal Motility Society 24: 503–512. 22583600 10.1111/j.1365-2982.2012.01921.x PMC5595063

[38] Barragan A, Sibley LD (2002) Transepithelial migration of Toxoplasma gondii is linked to parasite motility and virulence. J Exp Med 195: 1625–1633. 12070289 10.1084/jem.20020258 PMC2193562

[39] Barragan A, Brossier F, Sibley LD (2005) Transepithelial migration of Toxoplasma gondii involves an interaction of intercellular adhesion molecule 1 (ICAM-1) with the parasite adhesin MIC2. Cell Microbiol 7: 561–568. 15760456 10.1111/j.1462-5822.2005.00486.x

[40] Speer CA, Tilley M, Temple ME, Blixt JA, Dubey JP, et al (1995) Sporozoites of Toxoplasma gondii lack dense-granule protein GRA3 and form a unique parasitophorous vacuole. Mol Biochem Parasitol 75: 75–86. 8720177 10.1016/0166-6851(95)02515-4

[41] Treeck M, Zacherl S, Herrmann S, Cabrera A, Kono M, et al (2009) Functional analysis of the leading malaria vaccine candidate AMA-1 reveals an essential role for the cytoplasmic domain in the invasion process. PLoS Pathog 5: e1000322. 19283086 10.1371/journal.ppat.1000322 PMC2654807

[42] Donald RG, Carter D, Ullman B, Roos DS (1996) Insertional tagging, cloning, and expression of the Toxoplasma gondii hypoxanthine-xanthine-guanine phosphoribosyltransferase gene. Use as a selectable marker for stable transformation. The Journal of biological chemistry 271: 14010–14019. 8662859 10.1074/jbc.271.24.14010

[43] Brymora A, Valova VA, Robinson PJ (2004) Protein-protein interactions identified by pull-down experiments and mass spectrometry. Current protocols in cell biology/editorial board, Juan S Bonifacino [et al] Chapter 17: Unit 17 15. 10.1002/0471143030.cb1705s22 18228443

[44] Garrison E, Treeck M, Ehret E, Butz H, Garbuz T, et al (2012) A forward genetic screen reveals that calcium-dependent protein kinase 3 regulates egress in Toxoplasma. PLoS pathogens 8: e1003049. 23209419 10.1371/journal.ppat.1003049 PMC3510250

[45] Soldati D, Boothroyd JC (1993) Transient transfection and expression in the obligate intracellular parasite Toxoplasma gondii. Science 260: 349–352. 8469986 10.1126/science.8469986

[46] Donahue CG, Carruthers VB, Gilk SD, Ward GE (2000) The Toxoplasma homolog of Plasmodium apical membrane antigen-1 (AMA-1) is a microneme protein secreted in response to elevated intracellular calcium levels. Mol Biochem Parasitol 111: 15–30. 11087913 10.1016/s0166-6851(00)00289-9

[47] Battye TG, Kontogiannis L, Johnson O, Powell HR, Leslie AG (2011) iMOSFLM: a new graphical interface for diffraction-image processing with MOSFLM. Acta Crystallogr D Biol Crystallogr 67: 271–281. 21460445 10.1107/S0907444910048675 PMC3069742

[48] Evans PR (2005) Scaling and assessment of data quality. Acta Cryst D: 72–82. 10.1107/S0907444905036693 16369096

[49] Winn MD, Ballard CC, Cowtan KD, Dodson EJ, Emsley P, et al (2011) Overview of the CCP4 suite and current developments. Acta Crystallogr D Biol Crystallogr 67: 235–242. 21460441 10.1107/S0907444910045749 PMC3069738

[50] McCoy AJ, Grosse-Kunstleve RW, Adams PD, Winn MD, Storoni LC, et al (2007) Phaser crystallographic software. J Appl Crystallogr 40: 658–674. 19461840 10.1107/S0021889807021206 PMC2483472

[51] Schwarzenbacher R, Godzik A, Grzechnik SK, Jaroszewski L (2004) The importance of alignment accuracy for molecular replacement. Acta Cryst D60: 1229–1236. 10.1107/S0907444904010145 15213384

[52] Emsley P, Cowtan K (2004) Coot: model-building tools for molecular graphics. Acta Crystallog sect D 60: 2126–2132. 10.1107/S0907444904019158 15572765

[53] Murshudov GN, Vagin AA, Dodson EJ (1997) Refinement of macromolecular structures by the maximum-likelihood method. Acta Crystallog sect D 53: 240–255. 10.1107/S0907444996012255 15299926

[54] Burg JL, Perelman D, Kasper LH, Ware PL, Boothroyd JC (1988) Molecular analysis of the gene encoding the major surface antigen of Toxoplasma gondii. J Immunol 141: 3584–3591. 3183382

